# The role of educational initiatives in EFL teacher professional development: a study of teacher mentors’ perspectives

**DOI:** 10.1016/j.heliyon.2023.e13342

**Published:** 2023-02-04

**Authors:** Abdul Aziz Mohamed Mohamed Ali El Deen

**Affiliations:** a Department of English Language and Literature, College of Languages and Translation, Imam Mohammad Ibn Saud Islamic University (IMSIU), Riyadh, Saudi Arabia

**Keywords:** Language teacher, Teacher professional development, Educational initiatives, Teacher mentoring, TESOL

## Abstract

Teacher professional development (PD) is a continuous process through which teachers try to improve their pedagogical skills. Educational initiatives (a form of expert mentoring) represent one type of teacher PD practices. This study investigated the role of educational initiatives in improving language teacher professional growth from supervisors’ perspectives. The study focused on engaging a group of high school English-as-a-foreign-language (EFL) teachers in Egypt in a set of educational initiative activities with their mentors, and then asking the mentors to evaluate the teachers' PD objectives, pedagogical practice gains, and attitudes. Ten supervisors who acted as mentors and 30 English language teachers took part in the study. Using a quantitative observation sheet, the study measured the mentors' perceptions of the trainee teachers' target objectives from educational initiatives, their rating of the teachers' pedagogical performance after engaging them with these initiatives, and their evaluation of the teachers' attitudes towards their professional development. The study found differences in the mentors' perceived ratings of the teachers' professional interests, growth, and attitudes. Discrepancies were also noted within the observed aspects in the same dimension. The author discusses these results and provides some practical recommendations and suggestions for future research.

## 1 Introduction

There is always a need for teacher professional growth and for viewing it as a continuous process. Literature indicates there are two main approaches to teacher professional development (PD) [1,2,3,4,5]. These comprise the formal approach and the informal one. The formal approach to teacher PD includes the educational programs which centers on certain topics. As for the informal approach, it is usually self-initiated and self-directed. With this informal approach, the choice of training areas is relevant to and dependent upon teachers' needs and interests. Teacher educators can make use of such informal approach in helping teachers in fostering their PD.

Though many EFL teachers are keen on knowing about new teaching models and practices, they encounter certain barriers in their professional development (PD). These barriers could be eliminated by the instructors themselves [6]. Novozhenina and Lopez-Pinzon [7] reported that teachers in Colombia were usually loaded with extra work, such as preparation, planning and grading; this resulted in having inadequate time for updating their pedagogical knowledge. Likewise, teachers in Egypt experience a similar situation due to the same factor. According to Bor [8], there are problems related to language teachers when compared with teachers of other majors. These problems include: the need for outside support, the challenge of increasing subject knowledge and the absence of talented colleagues teaching the same subject. Unlike the teaching of other school subjects, foreign language pedagogy involves more challenging tasks such as teaching various language areas, developing the communicative competence in the target language, using a variety of language learning technological applications and tools, and understanding the target language culture and acquisition dynamics.

Reviewing research on the barriers to language teacher PD, Romero [9] concludes that some barriers relate to the teacher education programs focusing on transmitting teaching process and competence knowledge without paying due attention to enabling teachers develop these processes and competences. Similarly, many studies [10, 11, 12] have revealed other barriers impeding language teacher PD such as insufficient training, inadequate time, insufficient scaffolding from school's administration, structural challenges and strict hierarchical mandates. These results indicate the pressing need for structuring high quality language teacher PD programmes. Yates [13] argues that to achieve quality in teacher learning processes, continuing teacher education is prerequisite. In their study about Saudi primary school EFL teachers' professional training needs, Oudah and Altalhab [14] found that they need training delivered by qualified and professional trainers and educators. Therefore, there is a need for creating strong empirically-based evidence about instructor's continuous development with the aim of making well-informed policy decisions in future.

The status of the English-as-a-foreign-language (EFL) teacher PD in the Egyptian context is no exception. Some previous studies [15,16,17] have revealed similar barriers to EFL teacher PD in Egypt. Practically speaking, EFL teachers in Egypt attend short-term teaching training courses in which they are taught some rudimentary rules of English instruction. Research generally shows that EFL teachers in the Egyptian context are not satisfied with the training support and follow-up evaluation conducted with them [18]. Such short-term training does not seem to help them adopt current language pedagogy techniques. This case matches Cirocki and Farrell [19] view that many EFL teachers do not have adequate chances to develop their pedagogical skills. Accordng to Hismanoglu and Hismanoglu [20], many teachers need scaffolding from their supervisors or mentors while they are interacting with students to enhance their abilities and improve their pedagogical knowledge. Thus, the present study tried to fill in a gap in Egyptian EFL teacher PD through engaging a group of teachers in a set of educational initiative activities (a form of expert mentoring) with their mentors, and then asking the mentors to evaluate the teachers' PD objectives, pedagogical practice gains, and attitudes.

## 2 Literature review

### 2.1 Language teacher professional development and its importance

Researchers have proposed several definitions for teacher PD. Diaz-Maggioli [21] defines it as an ongoing learning process through which teachers get engaged willingly to enhance and learn how best to tailor their teaching processes to meet the learning needs of their students. PD is sought in a wide range of professions and businesses where teachers learn and apply new knowledge and skills that will improve their pedagogical performance. Ahmed, Mizell and Wong [17,22,23] view teacher PD as a lifelong attempt, a manner a teacher improves his/her being, and a perspective on how a teacher practices teaching as well as his teaching practice itself. On the other hand, Ahmed [17] also states that PD “may support teachers to be more responsible for planning and pursuing their ongoing learning, for reflecting with colleagues on their teaching practices” (p. 24). This view is consistent with Avalos's definition [24] of PD as “teachers' learning, learning how to learn, and transforming their knowledge into practice for the benefit of their students' growth” (p. 10).

Teacher education is a sustained and continuous growth process which is also known as Continuing Professional Development (CPD). Discussing the purposes of CPD, Day and Sachs [25] mention that they relate: “to align teachers' practice with educational policies, to improve the learning outcomes of the students by improving the performance of the teachers, or to enhance the status and profile of the teaching professional” (p. 22). In view of CPD tasks, teachers occasionally promote their perceptions about the effectiveness of self-regulated professional development. They practically experience the professional gains of self-driven activities and tasks which play the major roles in bringing about true enhancement to teachers’ pedagogical success and competence [26].

EFL/ESL teachers' promotion and training are considerably determined by their readiness to be involved in permanent reasoning and inquiry; such readiness represents the core of professional development. Inquiry-based professional development is defined as the teacher's continuing reflection and action on his/her teaching so as to find practical solutions to problems or issues facing him/her while teaching [27]. In-service teachers of English language are recommended to update, promote and enhance their teaching skills and their subject matter knowledge through professional development [28]. Villegas-Reimers also [29] states that enhancing teacher PD involves augmenting teaching effectiveness, and improving professional growth. Additionally, Darling-Hammond and Snowden [30] highlighted seven characteristics of effective professional development. Specifically, they state that it should be: “content focused; incorporates active learning utilizing adult learning theory; supports collaboration, typically in job-embedded contexts; uses models and modeling of effective practice; provides coaching and expert support; offers opportunities for feedback and reflection, and; is of sustained duration” [31] [pp. 5–6].

There are many factors that support teacher PD. One of these factors is reflection which is viewed as an essential element, a vital factor in teacher development processes. Saylag [32] thinks that “personal beliefs and reflections on professional development inform pedagogical choices” (p. 3851). Borg [33] emphasizes that teachers' beliefs guide their teaching practices. As a result, when teachers reflect on their teaching practices, they become more self-directed and adept to improve themselves. Ahmed [16] categorizes teacher reflection into three: technical reflection, practical reflection and critical reflection (p.40). For teachers to enhance their professional growth, they can go through reflection on the teaching action [34,35]. Thus, reflection is regarded as a crucial skill for teacher PD by a number of educationalists [17,36,37,38]. Research suggests that for effective teaching and learning to take place in the classrooms, teachers need to be reflective practitioners [39,40]. To do so, teachers need time to extend their understanding, analyze their learners' output, and adopt new strategies while teaching. This could occur through supervisors’ mentoring and guidance.

### 2.2 Educational initiatives and teacher education

One of the suggested attempts in activating and updating EFL teachers' continuing PD is the use of educational initiatives. As a form of expert mentoring, educational initiatives can be defined as an attempt exerted to learn new pedagogical ideas, make use of some teaching aids to facilitate and accelerate students' learning, enhance student literacy and create a positive and inclusive learning environment [41]. Accordingly, educational initiatives are a set of implementable actions and activities teachers use based on suggestions made by their mentors, and they are implemented via the teacher within the school in view of the direct guidance of the supervisors with the aim of making a change in the educational process to improve it, and to achieving particular desired goals. Such educational initiatives are important for providing an attractive learning environment, as well as stimulating teachers' motivation to teach and learn effectively. There are different types of educational initiatives that aim at improving the teaching practices of the English language, enhancing educational skills and abilities, and promoting students’ language skills. The educational initiatives in the current study concern a number of issues which include teaching methods, aids and technologies, and use of active learning strategies, feedback techniques and assessment tools.

Educational initiatives are crucial strategies for developing research skills and professional growth of EFL teachers. They help in raising trainees' achievement levels, and increase teacher professional growth. In addition, they create an integrated link between teachers, students, and administration in view of educational capabilities. Regarding the school environment, educational initiatives can develop the educational process. Educational initiatives have been theorized based on supervisors’ collaboration with EFL teachers in view of challenges they have faced in the classes. Thus, teachers participated themselves in suggesting and selecting such activities. Since EFL teachers represent a basic source for designing these initiatives, this PD approach is in line with many researchers [42] who reported that EFL teachers obtain high gains in pedagogical aspects when having the opportunity of selecting and assigning their PD activities.

Freeman [43] argues that language teacher pedagogy has long been unmapped in some regions due to classroom regular practices dominance. In the Egyptian context, EFL supervisors allocate a great deal of efforts in supporting in-service teachers. Such support is represented in several practices such as guiding, coaching, and mentoring. Mentoring initiatives have been emphasized in some studies. For example, Mann and Tang's study [44] revealed that while different mentoring practices supports novice teachers, the paucity of supervisors or mentors' observations and reflection impedes language teacher professional growth and development. Therefore, there is a need to investigate the impact of mentoring on Egyptian EFL teachers' perceptions and performance.

### 2.3 Mentoring and its impact on EFL teachers

Mentoring is an informal approach to teacher education. It generally means informal guidance and facilitative support, and it can be defined as a linear process of transferring knowledge from an experienced teacher to a less experienced one through conversations and reflection [45]. In other words, it is a collaborative activity in which novice teachers benefit from their experienced peers in solving instructional problems and minimizing the gap between theory and practice [46]. It is a process in which teachers receive support in their PD from trusted expert colleagues [47]. Wyatt and Dikilita [45] state that mentoring could also reduce the high attrition rates of novice teachers who feel unprepared for the teaching profession.

Some theories underpin the use of mentoring in teacher learning practices and PD. According to Nguyen [48], mentoring is based on the social construction theory of learning and the zone of proximal development (ZPD) perspective [49], and the reflective practitioner notion. The ZPD is defined as the difference between what a person has already known (actual development level) and what that person can fulfill with the guidance or collaboration opportunities involving more capable peers (Mann & Walsh, 2017 [50]). The ZPD supposes that for instruction to have a high rate of effectiveness, teachers need to engage learners in collaborative activities with more experienced peers through creating an interactive educational environment [51]. This perspective is regarded as the theoretical rationale for the mentoring process. Nguyen [48] explains on the role of social constructivism, the ZPD and reflection in teachers' mentoring as follows:

[The] ZPD provides a useful conceptual understanding of how teacher education programs can benefit from cooperative learning for those who are … in the process of learning how to teach. This idea of social constructivism underpins the need for opportunities for collaboration and support, and for learning. … Mentoring is rooted in the reflective practitioner tradition. Reflective practice is increasingly being recognised as important in teacher education. It is assumed that teachers learn from experience through continuous reflection on their teaching experience … Ideally, participants in the peer mentoring/mentoring process can apply one or more of these forms of reflection during their interaction with other teachers through a variety of mentoring strategies such as conversations, observation, discussions, lesson plan reviews, or collaborative work. (pp. 30-32).

Practicing mentoring in teaching English as a foreign language (TEFL) requires a set of requirements. These requirements include career progression from a job to another, years of experience, and the training received. Moreover, having unique qualifications and mastering academic, global, and digital skills are prerequisite to such task. The ultimate goal of mentoring is to direct teachers towards enhancing the teaching process [52]. Bailey [53] states that effective mentoring has some basic dimensions or levels, including the technical, collaborative, leadership, consultation, humanitarian and academic ones. When mentoring sessions occur, three phases should be reconsidered. According to Pawlas and Olivia [54], these three phases are: the pre-observation conference, the classroom observation, and lastly the post-observation conference.

EFL teachers can benefit from the mentors' visits to their classroom. Teachers have to devote more time for reading materials related to their pedagogical practices. Action research plays a very vital role in professional development. Elliot [55] describes educational action research as “an operation of reciprocal learning through which teachers learn from each other, and from their students as well as the latter learning from the former” (p. 182). In addition, another form of educational self-initiation teachers can adopt is to participate in specialized educational forums, seminars, webinars, conferences and workshops to benefit from others and to get acquainted with the new information useful to them in developing their performance. Teachers can also follow-up everything new in the field through the World Wide Web. Suwaed and Rohouma's study [56] indicates online reading sources helped their participant teachers develop professionally and to overcome the obstacles they encounter.

Some studies indicate that eachers' mentoring is effective in developing their pedagogical performance and perceptions. For example, Nabi Karimi and Norouzi [57] showed how novice teachers' pedagogical knowledge base could be enhanced as a result of expert mentoring initiatives. The mentoring initiatives in their study were represented in video-recorded performance analysis, expert-teacher observation and critical friendship. The novice teachers received such initiatives from the experienced ones. After a set of methodological procedures in their study, the results indicated significant differences before and after mentoring program in both the total frequency through which they showed pedagogical thought units and the relative dominance of pedagogical thought categories. This study has generally emphasized the important role of mentoring in enhancing teachers’ cognition development. In Vietnam, Nguyen and Tran [58] also found that pre-service EFL teachers had positive learning experiences as a result of mentoring in a practicum programme.

Teachers' mentoring has also been found beneficial to all parties concerned. For example, Borg and Parnham [47] found a number of benefits for teachers' mentoring in India. According to them, mentors developed increased confidence in supporting teachers, and better understanding of the relationship between instructional theories and classroom practices. As for the teachers, they had better confidence in their teaching interactively and developed their communication and reflection skills. The same gains were reported in another report [59]. In Turkey, Dikilitaş and Mumford [60] also showed how a three-week workshop mentoring programme was followed by a year-long programme of observations and feedback. Drawing on a previous study has positively influenced teachers and helped in developing greater trust between the teachers and their students, and in turn improving the teachers’ life quality and identity.

It can be noted that there is a scarcity of research on teachers' mentoring. In general, EFL teachers' mentoring is a research area that needs further studies, particularly in under-explored contexts such as Egypt. Therefore, the present study probed the role of mentor-led educational initiatives in improving the level of professional growth of EFL teachers. The importance of this study stems from the dire need most EFL teachers have for PD in a number of areas. Fostering these language teachers' PD could in turn positively influence their students' academic performance. Additionally, a few studies have explored language teacher PD from mentors’ perspectives. Expert mentors and teacher educators are able to crystalize key practical issues of utmost importance to teachers' PD [61]. This dimension adds to the importance of the present study.

## 3 The present study

Based on the above, the present study explored teacher mentors' perspectives on the role of educational initiatives in improving Egyptian EFL teachers' professional growth levels. In this study, educational initiatives were used to promote Egyptian EFL in-service teachers' PD and to help them overcome the pedagogical challenges they encounter. With this purpose, the study tried to fill in an important research gap many researchers have called for addressing [19, 59, 62]. Implementing teacher educational initiatives in this study was preceded by collecting pilot data from the supervisors about the teaching difficulties their supervisee EFL teachers encounter. Based on the pilot data collected, a set of educational initiatives were arranged for a group of teachers, and then the study explored these roles of these educational initiatives on the teachers' PD as perceived by their mentors.

The data for this study was collected from a group of English teaching mentors in Egypt using an observation sheet. Each participant mentor in this study was responsible for guiding three EFL teachers and providing them with adequate support for their PD, and then observing them in the classroom and evaluating their PD objectives and attitudes. Specifically, the study focused on the following three dimensions related to the teacher educational initiatives: the mentors' perceptions of the trainee teachers' target objectives from educational initiatives, their rating of the teachers' pedagogical performance after providing them with these initiatives, and their rating of the teachers' attitudes towards their PD. Accordingly, the present study tried to answer the following three research questions:

1. How do the mentors perceive the trainee teachers' target objectives from educational initiatives?
2. How do the mentors rate the trainee teachers' pedagogical performance after providing them with educational initiatives?
3. How do the mentors rate the trainee teachers' attitudes towards their PD after providing them with educational initiatives?

### 3.1 Research procedures

The author followed the four main steps below in conducting the study:

First: identifying teachers' mentoring needs. This has been accomplished through interviewing five supervisors and 10 teachers about these needs. The interviews were held electronically via Zoom and focused on getting data about the teachers' mentoring needs. Following this, the author identified the teachers' mentoring needs through analyzing the notes taken during the interviews.

Second: identifying the mentoring objectives and guidelines, and developing teacher evaluation instrument. These objectives and guidelines were identified based on the results of the teacher and supervisor interview data.

Third: Implementing teacher training and mentoring sessions. Each supervisor was assigned three teachers to monitor and guide in four sessions, two sessions in each week. These sessions were guided by the objectives and guidelines identified by the author. These include: getting teachers to talk about their teaching weaknesses and their PD objectives, and developing their attitudes and awareness towards PD.

Fourth: Evaluating teachers' professional interests, growth, and attitudes. This stage involved using the teacher evaluation instrument developed by the author in assessing the teachers' professional interests, growth, and attitudes. The supervisors completed the sheet pertaining to each teacher based on visiting him two times in the week following the mentoring sessions, and also in light of observing their interests and attitudes in the mentoring sessions and in the post-observation discussion.

### 3.2 Setting and participants

This study was conducted in high schools in Sohag governorate, Egypt. All the mentors and teachers who took part in the study were all males working in the field of English language education in the district. Ten supervisors participated in this study. Each supervisor who acted as a mentor in this study provided three teachers with professional guidance and support. Thus, in total 10 supervisors and 30 teachers took part in the present study. Six supervisors had 15 years of supervision work experience, and four had 12 years of supervision experience. Five supervisors had a postgraduate diploma in TESOL, and the other five had a BA degree. They were all in their fifties during the data collection stage. Initial background interviews with the supervisors indicate they all had a great deal of academic, digital, administrative, and mentoring competence acquired through training sessions and workshops. As for the teachers, they all had a BA degree and their ages ranged from 24 to 46 years. They were working in 6 high schools in Sohag governorate. Informed consent was obtained from all the supervisors and teachers who took part in this study voluntarily.

### 3.3 Teacher evaluation instrument

In light of critically reviewing pertinent literature on language teacher professional development, a 20-item observation sheet was prepared for identifying EFL supervisors’ of the teachers' responses to the educational initiatives. The author developed the observation sheet guided by the pilot data gathered from the five supervisors and 10 teachers about the teachers' PD needs. The final version of the observation sheet includes three parts about the above three dimensions, i.e., namely, the mentors' perceptions of the trainee teachers' target objectives from educational initiatives, their ratings of the teachers' pedagogical performance after providing them with these initiatives, and their ratings of the teachers' attitudes towards their PD. Each dimension has a number of items (i.e., the first dimension has six items; the second one has seven items, and the third one has seven items, as well) see Table 3, Table 4, Table 5. The observation sheet has a 5-point Likert scale (5 = strongly agree, 4 = agree, 3 = neutral, 2 = disagree, and 1 = strongly disagree).

To ensure the validity of the observation sheet, it was read by two expert language education researchers who evaluated it and confirmed the relevance of the statements to the domains and appropriateness of the linguistic formulation of its items. As for the reliability of the observation sheet, it was evaluated using Cronbach's alpha to determine the internal consistency its items and sections. The statistical analyses showed that the observation sheet as a whole has a reliability coefficient of 0.91. Table 1 shows the reliability coefficients of the observation sheet and its parts.

**Table 1:** Reliability coefficients of the dimensions covered in the observation sheet.

Dimension of the observation sheet	Cronbach's alpha coefficient
The first dimension	0.62
The second dimension	0.80
The third dimension	0.86
Total scale	0.91

### 3.4 Data collection and analysis procedures

The data of this study was collected in two weeks. The author obtained the institutional approval for collecting data prior to approaching the participants. After each supervisor provided mentoring and guidance for three teachers over two weeks, they observed them in their classes two times and discussed with these issues related to the measured dimensions. Then, each supervisors completed the observation sheets related to the three teachers they were assigned to. The author received the observation sheets of the 30 teachers over two weeks.

The data was analyzed using descriptive statistics. The means of the supervisor's ratings of each teacher in the two observation sheets were calculated, and then the overall mean for the 30 teachers was obtained. Utilizing the descriptive method, the percentages, means and standard deviations were calculated for the items related to the three dimensions of the mentors' observation. For statistical analysis, the relative weight of each item was categorized into five levels: very poor, poor, medium, high, and very high. Table 2 shows the mean degrees of initiative for each of these five levels.

**Table 2:** The weight criterion of the items in the observation sheet.

Mean		Degree of Initiative
From	to	
1	1.8	Very poor
1.9	2.6	Poor
2.7	3.4	Medium
3.5	4.2	High
4.3	5	Very high

## 4 Results of the study

In this section, the author presents the results of the current study in three sections. Each section is related to one dimension covered in the observation sheet.

### 4.1 Teachers' target objectives from educational initiatives

The first research question relates to the mentors' perceptions of the trainee teachers' target objectives from educational initiatives. This question was answered by analyzing the supervisors' evaluations in the first part of the observation sheet. Table 3 gives the means, standard deviations, and degrees of initiative for each statement in this part and also for the whole part. As the table shows, the teachers' target objectives from educational initiatives have an overall high mean (4.16). The first item (i.e., acquiring some new skills and experiences) has the highest mean with a very high initiative degree. This outcome is confirmed by Bailey et al. [63] who explain the reason why teachers endeavor to attend activities designed for professional development is that they gain new knowledge and enhance their teaching skills. Moreover, the results of Karataş and Tuncer's study [64] emphasize that process-oriented activities, collaboration, cooperation, and interaction among pre-service EFL teachers, in virtual contexts, can facilitate the development of their language skills. Thus, the present study is congruent with Bailey et al.‘s study [63] findings with regard to the first statement in the first dimension. The high degree of the initiative of the first statement reflects EFL teachers' strong desire in acquiring new skills and experiences.

**Table 3:** The mentors' perceptions of the trainee teachers' target objectives from educational initiatives.

Statement		Mean	Standard deviation	Degree of Initiative
The teachers' discussion in the mentoring sessions focuses on:				
1.	Acquiring some new skills and experiences.	4.53	0.57	very high
2.	Developing students' language proficiency.	4.20	0.48	High
3.	Improving students' language skills.	4.07	0.52	High
4.	Creating a positive learning environment.	4.10	0.61	High
5.	Supporting good relationships with colleagues.	4.27	0.69	High
6.	Investing free time by practicing educational initiatives.	3.80	0.48	High
	Total score	4.16	0.56	High

The mean initiative degree in the second statement is high (M = 4.20). This emphasizes Selim's (2009) [65] view that language proficiency is one of the prominent factors in preparing EFL instructors and developing students' proficiency. Language proficiency has been claimed to be a significant aspect contributing to students' academic learning performance (Martirosyan, Hwang, & Wanjohi, 2015 [66]). This high mean is also congruent with Wang's view [67] that the importance of EFL teachers' proficiency in the enhancement of learners' language performance. The initiative degree of the third statement three is 4.07. This indicates the importance of language skills in making students master English language learning. Jenkins [68] asserts that English teaching and learning thrive when students are able to proficiently practice language skills inside and outside the classroom. Thus, the current study supports Jenkins' view about the important role of the teacher in helping students master language areas.

The fourth statement (i.e., creating a positive learning environment) has also a high mean initiative degree. Mutlu and Yıldırım's research findings [69] indicate that the features of EFL learning environment and student background variables were significantly related to student determination in EFL learning. Meanwhile, the mean degree of initiative in the fifth statement (i.e., supporting good relationships with colleagues) is high. This does not concur with Cowie's (2011) [70] study which showed that the teachers had very positive feelings of emotional. As for the teachers' aim to invest free time in practicing educational initiatives (i.e., the sixth statement), it has a mean of 3.80 which generally shows the value of free time in improving teaching environment. Other studies showed that emergency distance education has created leisure time that teachers could not have when teaching in face-to-face classes. Accordingly, teachers could spend this free time in developing their language skills and teaching practices [64].

### 4.2 Teaching practices in EFL classrooms

The second part of the observation sheet is concerned with the mentors' rating of the trainee teachers' pedagogical performance after engaging them in educational initiatives. This part relates to the second research question. Table 4 shows the means, standards deviations, and the degrees of initiatives of the statements in this part.

**Table 4:** The mentors' rating of the trainee teachers' pedagogical performance after engaging them in educational initiatives.

Statement		Mean	Standard deviation	Degree of Initiative
The teacher uses:				
7.	Recent teaching methods.	3.57	1.07	High
8.	Suitable teaching aids.	3.00	0.59	Medium
9.	The available teaching technologies.	3.80	0.61	High
10.	Active learning strategies.	3.57	0.90	High
11.	Different techniques to help student participate in activities.	3.27	0.74	Medium
12.	Feedback techniques to enhance learning.	3.33	0.55	Medium
13.	Different assessment tools.	3.10	0.48	Medium
	Total score	3.38	0.70	Medium

As noted in the table, the seventh statement (i.e., using recent teaching methods) has a high mean of 3.57. Almuhammadi [71] points out educational institutions should conduct periodical professional development courses in order to equip EFL teachers with up-to-date methods to teach English grammar properly. With the rapid changes and technology dominance in teaching English, EFL instructors have had a pressing need for adopting recent teaching methods. The eighth statement (i.e., using recent teaching aids) has a medium mean and degree of educational initiative. Ahmed [15] views that teachers' avoidance of the instructional aids use could negatively affect students’ motivation. Therefore, teachers should assign and create their own teaching aids for making teaching and learning more effective. Regarding the ninth item, it has a high degree of initiative, and a mean of 3.80. Research shows that in some contexts where EFL teachers' information and communication technology (ICT) competence is low, it is necessary to provide them with a medium and higher amount of ICT training [72].

The overall degree of initiative for this second dimension has a medium rank. One of the important teaching practices relate to active learning strategies. In this regard, the tenth statement (i.e., using active learning strategies) has a high degree of initiative of 3.57. Various studies indicate that burgeoning theories to the educational process can be carried out in learning English language via applying recent applications of computer-assisted language learning [73]. This emphasizes the need for depending on technology-mediated learning tools, including: mobile-assisted language learning (MALL), and language massive open online courses (LMOOCs).

Statement 11 (i.e., using different techniques to help student participate in activities) has a medium mean of 3.27. This can be ascribed to the much scientific reasoning and knowledge teachers are likely to draw upon in interacting with learners using meaningful activities. Likewise, the degree of initiative for statement 12 (i.e., using feedback techniques to enhance learning) has a medium mean of 3.33. Numerous studies indicate that the teachers should give feedback focusing on high-order skills i.e. organization of contents, coherence, development of ideas etc. Than lower-order ones (i.e. grammar, sentence structure) [74,75]. Similarly, statement 13 (i.e., using different assessment tools) has a medium mean of 3.10. It is generally noted that many EFL instructors are not aware of alternative assessment techniques. This may have been caused by the large class sizes and the deficient professional training programs.

### 4.3 Supervisors' rating of the teachers' attitudes towards their professional development

The third part in the observation sheet concerns the mentors' rating of the teachers' attitudes towards their PD. Table 5 shows the means, standard deviations, and degrees of initiative of the items in this part. As noted, statement 14 (i.e., teachers' keenness to attend workshops and training courses) has a medium mean and initiative degree. McKinney et al. [76] point out that PD programs create opportunities for teacher learning and provide practical experience such as workshops, meetings and informal experience that the teachers accumulate as part of their work. The medium initiative degree of this statement could mean that some EFL teachers in Egypt are not interested in attending PD training programmes. This could have resulted from many economic factors and teachers’ low social status [77].

**Table 5:** The mentors' rating of the teachers' attitudes towards PD.

Statement		Mean	Standard deviation	Degree of initiative
14.	The teacher is keen to attend workshops and training courses.	3.27	0.58	Medium
15.	The teacher is aware of self-development.	3.10	0.40	Medium
16.	The teacher participates in online or self-paced courses or programs.	3.37	1.00	Medium
17.	The teacher engages in informal dialogues with colleagues on how to improve teaching.	2.77	0.68	Medium
18.	The teacher makes use of the feedback for improving certain aspects of his work.	2.60	0.81	Poor
19.	The teacher participates in discussions about how to improve teaching.	2.57	0.86	Poor
20.	The teacher makes use of action research for mutual learning.	2.60	0.93	Poor
	Total score	2.90	0.75	Medium

Statement 15 (i.e., teachers' awareness of self-development) has a medium initiative degree with mean of 3.10. This result goes in line with Rachmajanti et al.‘s study [78] which revealed that language teachers were subconsciously aware that teaching competence is undeniable for the sake of students' progress in learning English. Alshaikhi [79] also found that many participants highly preferred self-directed learning to institutional provisions for its real relation to their context, the nature of their specialization, and for the changing nature of their profession. Statement 16 (teachers' participation in online or self-paced courses or programs) has a medium mean of 3.37. Truong and Murray's study [80] confirms the vital role of information technology in online teacher professional development, and added well-established social norms that online training courses need to include to be more broadly accepted by EFL teachers.

With regard to statement 17 which relates to teachers' engagement in informal dialogues with colleagues on how to improve teaching, its mean is 2.77 and it has a medium educational initiative level. Afshar and Ghasemi [81] suggest that professional development policy makers have to plan effective and durable PD activities such as peer observation, teacher study groups, social media-based programs and online teacher PD methods in order to interest and involve EFL teachers in up-to-date PD activities. Statement 18 has a low mean of 2.60, indicating the teachers' poor abilities to make use of feedback forms. In their study about EFL teachers' professional development, Zhiyong et al. [82] recommend that teachers' reflection should be treated as an obligatory prerequisite for the teachers’ annual evaluation to guarantee the continuous professional development of EFL teachers.

As for statement 19, its low mean indicates that the teachers do not adequately participate in discussion their PD. Developing communication skills is essential characteristic for 21st century EFL teachers [83]. The development of communication skills involves advancement of active listening and proper responding, promoting the ability for delivering oral presentations, improvement of successful engagement in discussions and development of the ability for identifying and respecting others' attitudes, behaviors and beliefs [84,85]. Finally, statement 20 has a low mean of 2.60 and a poor degree of initiative. This means that many of the teachers do not incline to use action research in their classes or perhaps they are unaware of action research. Other researchers found that English language teachers neglect reflective teaching due to the more work load it requires [78]. The results of Van's study [86] showed that despite the recognized importance of action research, as a form of PF, there were a number of difficulties encountering its implementation.

## 5 Discussion of the results

As noted in the above results of the study, there is a discrepancy among the dimensions of the teachers' educational initiatives. While the observation sheet as a whole has a medium initiative degree, the mean degrees for the first and second dimensions are high and medium, respectively. Such discrepancy in the mean degrees of the dimensions may be due to some factors, including the scarcity of training programs available for EFL teachers' professional development in this context. Another factor is the inadequacy of pedagogical knowledge that is crucial for EFL teachers. In turn, this has caused a medium initiative degree in the second and the third dimensions, on one hand, and a poor degree of initiative within the items in a separate way on the other hand. Regarding the dimensions of teaching practices and teachers’ training, their medium initiative degrees indicate students' inadequacies in this dimension. As noted, the first dimension (i.e., mentors' perceptions of the trainee teachers' target objectives from educational initiatives) has a high mean as a whole and in its individual items. The second dimension (i.e., the mentors' rating of the trainee teachers' pedagogical performance after engaging them in educational initiatives) has a medium degree as a whole, although, the degrees of the initiative in some of its items are high. On the contrary, the degree of initiative is either medium or poor in the statements of the third dimension. This might be due to the fact that many EFL teachers in Egypt are not fully interested in the PD issues this dimension is concerned with such as using different types of feedback such as clarification, recasts, meta-linguistic feedback, elicitation and other types, or employing action research.

Regarding the first dimension as a whole and the statement of acquiring new skills in particular, the results of the present study are consistent with Zaghar's study [87] which asserts that professional development for teachers is implied in acquiring new skills, and finding a powerful learning environment. When compared to the target objectives from educational initiatives, the mentors' ratings of the teachers' classroom practices and interest in PD have a lower initiative degree. Although the findings indicate that EFL teachers are aware of the importance of educational initiatives for their PD, the medium degree of initiatives, noted in teachers' classroom practices and interest in PD could be attributed to the lack to their prioritized PD educational initiatives.

The present study indicates that educational initiatives represent one of the proactive constructs that should be entrenched in language pedagogy. Emphasizing the necessity of these initiatives in EFL/ESL settings, Nabi Karimi and Norouzi [57] state that directing more capable instructors to share a great deal of experience with beginning teachers could be a more economical attempt to make novice teachers realize their potential abilities. The current study is in line with Hismanoglu's study [88] which revealed that only 30% of the teachers paid due attention to CPD through utilizing the identified strategies, such as participatory practitioner research, PD portfolios, and study groups. However, Hismanoglu concludes that the most teachers were aware of the pressing need for PD in his study. This study goes in line with Day's study [89] in emphasizing the factors influencing EFL teachers' professional development and creating a moderate degree in the initiatives.

The results of the present study go in line with Wyatt and Dikilita's view [45] that mentoring helps teachers function successfully. These results also emphasize the importance of social construction and collaborative learning environments [49] in developing teachers' performance. They also support the previous research findings that mentoring can help EFL teachers improve their pedagogical performance [47, 57, 58, 59, 60] However, it is worth noting that both the present study and these previous studies indicate that for mentoring to be more effective, mentors' role is fundamental in preparing EFL teachers and enabling them to perform various pedagogical tasks, and overcome many challenges they encounter. Contextual issues should also be overcome to help teachers have the desired gains from mentoring.

## 6 Conclusions

The present study has explored the role of mentoring in improving EFL teachers' PD orientations and pedagogical practices. A group of Egyptian EFL teachers were engaged in a set of educational initiative activities with their mentors, and then they were observed. As noted in the results, the teachers have generally developed better orientations towards their PD, and improved particular aspects in their classroom performance. However, the mentors' ratings of the trainee teachers' performance and perceptions showed discrepancies in some observed aspects within the same dimension. Such discrepancies are attributed to some factors such as inadequate training, lack of teachers' interest, and prioritized PD initiatives.

One of the important points raised by the current study is the possibility of categorizing the role of educational initiatives as an informal approach to language teacher PD. This issue is also noted in the classification of approaches to PD [1, 2, 3, 4, 5]. These researchers state that the formal approach draws upon workshops and instructional programs, while the informal one focuses heavily on supervisors' or mentors’ help and guidance. In other words, the informal approach represents a model of self-initiation and self-guidance. In light of the study findings, the author recommends promoting and sharing experiences between the faculties of education and languages in universities and the supervisors of the Ministry of Education through educational meetings and conferences. This could help in preparing the future teachers who will be responsible for developing the educational process in the future. Recommended also is promoting electronic communication between supervisors and teachers. There is also a need for expiating all the necessary materials in order to produce an encouraging environment for supervisors to help them monitor and guide instructors who can produce creative initiatives.

This study is limited in collecting its final data from teachers' mentors only; teachers' needs were only assessed in the pilot stage. Therefore, future research on teacher educational initiatives could draw upon gathering final assessment data from both teachers and mentors. The study is also limited by the nature of the geographical region from which its data was collected. Therefore, future studies may examine how teachers in other parts of Egypt respond to this type of educational initiatives. Another limitation was the use of one source for collecting data. More research is needed to confirm these results and also to explore other types of educational initiatives such as extra curricula activities. Moreover, future research could also investigate the nature of continuing professional development activities with more participants and with triangulation of data, as well. More empirical studies are needed to examine the role of educational initiatives in enhancing EFL instructors’ PD in other Arab countries.

## Declarations

### Author contribution statement

Abdul Aziz Mohamed Ali El Deen, Ph.D: Conceived and designed the experiments; Performed the experiments; Analyzed and interpreted the data; Contributed reagents, materials, analysis tools or data; Wrote the paper.

### Funding statement

This research did not receive any specific grant from funding agencies in the public, commercial, or not-for-profit sectors.

### Data availability statement

Data will be made available on request.

### Declaration of interest's statement

The authors declare no conflict of interest.
